# Application of Metabolomics and the Discovery of Potential Serum Biomarkers for Diuretic Resistance in Heart Failure

**DOI:** 10.31083/RCM27001

**Published:** 2025-04-22

**Authors:** Yipin Yu, Qiong Yi, Chenglong Yang, Xudong Song, Duoting Tan, Qinghua Peng, Xiang Sun, Hao Liang

**Affiliations:** ^1^Institute of TCM Diagnostics, Hunan University of Chinese Medicine, 410208 Changsha, Hunan, China; ^2^ICU Department, The First Hospital of Hunan University of Chinese Medicine, 410021 Changsha, Hunan, China; ^3^Cardiovascular Department, The First Hospital of Hunan University of Chinese Medicine, 410021 Changsha, Hunan, China; ^4^Hunan Provincial Key Laboratory of TCM Diagnostics, Hunan University of Chinese Medicine, 410208 Changsha, Hunan, China; ^5^Cardiology Department, Changsha Hospital of Chinese Medicine, 410001 Changsha, Hunan, China

**Keywords:** heart failure, diuretic resistance, metabolomic, biomarker

## Abstract

**Background:**

Diuretic resistance (DR) is characterized by insufficient fluid and sodium excretion enhancement despite maximum loop diuretic doses, indicating a phenotype of refractory heart failure (HF). Recently, metabolomics has emerged as a crucial tool for diagnosing and understanding the pathogenesis of various diseases. This study aimed to differentiate diuretic-resistant patients from non-resistant HF to identify biomarkers linked to the emergence of DR.

**Methods:**

Serum samples from HF patients, both with and without DR, were subjected to non-targeted metabolomic analysis using liquid chromatography-tandem mass spectrometry. Metabolite variations between groups were identified using principal component analysis and orthogonal partial least-square discriminant analysis. Metabolic pathways were assessed through the Kyoto Encyclopedia of Genes and Genomes database enrichment analysis, and potential biomarkers were determined using receiver operating characteristic curves (ROCs).

**Results:**

In total, 192 metabolites exhibited significant differences across the two sample groups. Among these, up-regulation was observed in 164 metabolites, while 28 metabolites were down-regulated. A total of 28 pathways involving neuroactive ligand-receptor interaction and amino acid biosynthesis were affected. The top five metabolites identified by ROC analysis as potential DR biomarkers were hydroxykynurenine, perillic acid, adrenic acid, 5-acetamidovalerate, and adipic acid.

**Conclusions:**

Significant differences in metabolite profiles were observed between the diuretic-resistant and non-diuretic-resistant groups among patients with HF. The top five differentially expressed endogenous metabolites were hydroxykynurenine, perillic acid, adrenic acid, 5-acetamidovalerate, and adipic acid. The metabolic primary pathways implicated in DR were noted as amino acid, energy, and nucleotide metabolism.

**Clinical Trial Registration:**

This study was registered with the China Clinical Trials Registry (https://www.chictr.org.cn/hvshowproject.html?id=197183&v=1.7, ChiCTR2100053587).

## 1. Introduction

Heart failure (HF) is a complex clinical syndrome caused by structural and/or functional abnormalities within the heart, characterized by the heart’s inability to pump sufficient blood and oxygen to meet the metabolic demands of other organs. The World Health Organization estimates that approximately 64.3 million individuals, constituting 1% to 2% of the global population, are afflicted by HF. Overactivation of the sympathetic and renin-angiotensin-aldosterone systems (RAAS) leads to water and sodium retention, resulting in extracellular volume expansion and a significant deterioration in a patient’s condition [1, 2]. Key symptoms such as lung congestion, peripheral oedema, and elevated jugular venous pressure are commonly seen in patients with HF [3]. Consequently, diuretics serve as fundamental therapeutic agents aimed at alleviating symptoms and signs attributable to water and sodium retention [4, 5, 6].

As the frequency and dosage of diuretic administration increase, the effectiveness of diuresis diminishes, leading to the development of diuretic resistance (DR). DR can be succinctly defined as either a diminished or complete absence of response to loop diuretics [7]. Specifically, a commonly used measure is the fractional excretion of sodium. The fractional excretion of sodium refers to the proportion of the filtered sodium load that is excreted from the body in the form of sodium (mmol/time) [8]. DR is present when this fraction is less than 0.2. Under normal physiological conditions, the kidneys filter the sodium in the blood, reabsorb part of the filtered sodium as needed, and excrete the rest in the urine. When loop diuretics are used, they normally promote sodium excretion and increase the fractional excretion of sodium. However, in the case of DR, even if loop diuretics are administered, the fractional excretion of sodium remains below 0.2%, indicating that the diuretic’s effect of promoting sodium excretion has not been effective. Approximately one-third of patients experience DR [9], characterised by an inability to enhance renal sodium and water excretion through diuretic therapy, resulting in persistent symptoms of volume overload and edema [5, 10, 11]. The DR is a refractory phenotype in the progression of HF, often necessitating frequent hospital admissions and stays in the intensive care unit. It is independently associated with worsening renal function and death [12, 13, 14]. Diagnosis of DR typically occurs after a patient’s non-responsiveness to escalated diuretic doses, often with a considerable time lag. Therefore, identifying reliable biomarkers for the timely detection of DR is crucial. This could facilitate prompt intervention and potentially improve the prognosis.

Introduced by Professor Nicolson in 1999, metabolomics has emerged as a crucial element within the realm of systems biology. The primary objective of this analysis is to discern the relative associations between metabolites and pathological alterations [15]. With advancements in technology and metabolic databases, metabolomics can uncover insights into cardiovascular disease and identify potential new biomarkers [16, 17, 18]. In patients with HF, changes in circulating metabolites reflect metabolic alterations in both the heart and peripheral tissues, with these peripheral metabolic changes being an integral part of the pathogenesis and disease progression of HF [19, 20]. Therefore, this prospective study used metabolomics techniques to identify differential metabolites as biomarkers for diagnosing DR in HF.

## 2. Methods

### 2.1 Population 

Participants were recruited from the First Affiliated Hospital of Hunan University of Chinese Medicine and the Changsha Hospital of Chinese Medicine between December 2021 to December 2022. The study was approved by the Ethics Committee of The First Affiliated Hospital of Hunan University of Chinese Medicine under the ethical approval number HN-LL-SZR-2021-10. It was registered with the China Clinical Trials Registry (https://www.chictr.org.cn/hvshowproject.html?id=197183&v=1.7, ChiCTR2100053587), and written informed consent was obtained from all participants prior to their involvement in the study.

### 2.2 Inclusion and Exclusion Criteria

Inclusion criteria included individuals aged between 50 and 85 years who met the diagnostic criteria [21] for HF according to the 2018 Guidelines for the Diagnosis and Treatment of HF in China, along with having a New York Heart Association (NYHA) functional class of ≥Grade III.

Exclusion criteria included individuals eligible for hemodialysis, those with a systolic blood pressure of ≤80 mmHg, a glomerular filtration rate of ≤15 mL/min, serum albumin levels of ≤2.5 g/dL, serum potassium levels of ≥5.5 mEq/L, serum sodium levels of >145 mEq/L, and anuric patients with a urine volume of ≤100 mL per 24 hour (h). In addition, individuals with a history of myocardial infarction or unstable angina within the past 3 months or those who underwent coronary revascularization (either surgical bypass surgery or angioplasty) were excluded. Individuals with severe primary diseases such as those affecting the hematopoietic system or malignant tumours were also excluded.

### 2.3 Identification of DR and Baseline Characteristics

To elucidate the crucial physiological process of the diuretic response, the initial treatment with loop diuretics was initiated immediately upon the patients’ admission. The changes in urine volume and body weight, which can effectively reflect the diuretic effect, were examined. The drug were standardized, with dosages equivalent to 48 h/40 mg of furosemide [13, 22]. After treatment, the patient’s urine volume change of <+1000 mL/48 h/40 mg of furosemide or a weight change of ≥+0 kg/48 h/40 mg of furosemide, indicated the presence of DR if either condition was met [23]. This definition is based on the fact that if the urine volume does not change, it indicates that the diuretic fails to effectively promote urine excretion. Additionally, if the body weight does not decrease or even increases, it indicates that the fluid retention in the body has not improved. Both situations imply that the patient has not shown the expected response to the treatment. Finally, 60 patients, comprising 30 with DR and 30 without DR, met the inclusion criteria and were enrolled in the study.

The baseline characteristics of the patients were the first valid values recorded after hospital admission. Information such as coronary heart disease, cardiomyopathy, and atrial fibrillation were obtained from the history diagnosis results in the electronic medical record system. The body mass index (BMI) was calculated using the following formula: BMI = weight (kg)/height (m^2^). The estimated glomerular filtration rate (eGFR) was calculated using the 2021 Chronic Kidney Disease Epidemiology Collaboration (CKD-EPI) formula [24]: eGFR [mL/(min × 1.73 m^2^)] = 142 × (Scr/A)^B^ × (0.9938)^age^ × C (Scr: serum creatinine).

For females: The value of C is 1.012.

When Scr ≤0.7 mg/dL, A = 0.7 and B = –0.241.

When Scr >0.7 mg/dL, A = 0.7 and B = –1.2.

For males: The value of C is 1.

When Scr ≤0.9 mg/dL, A = 0.9 and B = –0.302.

When Scr >0.9 mg/dL, A = 0.9 and B = –1.2.

### 2.4 Sample Size Estimation

Statistical efficacy and sample size for the *t*-tests were calculated using https://www.statskingdom.com/sample_size_t_z.html. The results indicated that a minimum of 26 samples per group were necessary (with α = 0.05 and effect size = 0.8), as depicted in the **Supplementary File 1**.

### 2.5 Sample Collection

Blood samples were collected in the morning after determining whether the patients were diuretic-resistant or non-diuretic-resistant. Subsequently, they were allowed to stand at room temperature for 1 h before undergoing centrifugation at 3000 rpm for 15 min (HT230R, Xiangyi Experiment Equipment Co., Ltd., Changsha, China). The resulting clear supernatant was transferred into a 1.5 mL Eppendorf tube and refrigerated at –80 °C (DW-86W100, Haier, Qingdao, China) until further analysis.

### 2.6 Metabolomics Analysis

#### 2.6.1 Sample Preparation 

The samples were taken out from the –80 °C freezer and thawed at 4 °C. After thawing, each sample was vortexed for 1 minute to ensure complete mixing (BE-2600, Kylin-bell Lab Instruments Co., Ltd., Haimen, China). Then, an accurate volume of the sample was transferred into a 2 mL centrifuge tube. Subsequently, 400 µL of methanol (67-56-1, Fisher Scientific, Loughborough, UK) (stored at –20 °C) was added to the tube, and the mixture was vortexed again for 1 minute. Next, the sample was centrifuged at 12,000 rpm and 4 °C for 10 minutes. The resulting supernatant was carefully transferred to a new 2 mL centrifuge tube, concentrated, and dried. Finally, 150 µL of a 2-chloro-l-phenylalanine (103616-89-3, Aladdin, Shanghai, China) (4 ppm) solution, which was prepared with 80% methanol - water (stored at 4 °C), was added to redissolve the sample. The supernatant was filtered through a 0.22 µm membranem (Tianjin Jinteng Experiment Equipment Co., Ltd., Tianjin, China) and transferred into a detection bottle for liquid chromatography (LC) - mass spectrometry (MS) analysis [25].

#### 2.6.2 Liquid Chromatography 

The LC analysis was performed using a Vanquish UHPLC System (Thermo Fisher Scientific, Waltham, MA‌‌, USA). For LC-electrospray ionization (ESI) (+)-MS analysis, the mobile phases consisted of formic acid (64-18-6, TCI, Shanghai, China) in acetonitrile ((75-05-8, Fisher Scientific, Loughborough, UK) and formic acid (64-18-6, TCI, Shanghai, China) in water (Millipore, Bedford, MA, USA)). LC-electrospray ionization (ESI) (-)-MS analysis involved the use of acetonitrile (75-05-8, Fisher Scientific, Loughborough, UK) and ammonium formate (540-69-2, Sigma-Aldrich, Shanghai, China) (5 mM). Detailed information is included in the supplementary methods [26].

#### 2.6.3 Mass Spectrometry 

Metabolite detection via MS was performed using Orbitrap Exploris 120 (Thermo Fisher Scientific, Waltham, MA‌‌, USA) with an ESI ion source. The acquisition method employed simultaneous MS1 and MS/MS (full MS-ddMS2 mode, data-dependent MS/MS). Parameters included MS/MS resolving power set at 15,000 FWHM, normalised collision energy at 30%, and dynamic exclusion time set to automatic. Detailed information is included in the supplementary methods [27].

### 2.7 Data Analysis

#### 2.7.1 Data Processing and Annotation 

The raw data underwent initial conversion to the mzXML format using MSConvert within the ProteoWizard software package (v3.0.8789) [28], followed by processing using XCMS [29] for feature detection, retention time correction, and alignment. Metabolite identification relied on accurate mass (<30 ppm) and MS/MS data, which were matched with databases such as the Human Metabolome Database [30], MassBank [31], LipidMaps [32], mzcloud [33], and the Kyoto Encyclopaedia of Genes and Genomes (KEGG) [34]. To ensure accuracy, robust locally estimated scatterplot smoothing signal correction [35] was employed for data normalisation, effectively correcting for any systematic bias. Following normalisation, only ion peaks with relative standard deviations <30% in quality control (QC) were retained to ensure accurate metabolite identification.

#### 2.7.2 Statistical Analysis and Visualisation 

R software (ver. 4.2.1, The R Foundation, Vienna, Austria) was used for statistical analysis. The sample data was analysed for dimension reduction through principal component analysis (PCA) and orthogonal partial least-square discriminant analysis (OPLS-DA) with the Ropls package [36]. The data was scaled to show the differences in metabolite composition among samples (Q2 >0.5; R2 >0.5). All models were checked for overfitting through permutation tests. OPLS-DA helped to identify discriminative metabolites by using the variable importance on projection (VIP) metric. The *p*-value, VIP score, and fold change were used to identify contributing variables for classification. Pathway analysis of differential metabolites was carried out using MetaboAnalyst (McGill University, Quebec, Canada) [37], which combines robust pathway enrichment analysis with pathway topology analysis. The identified metabolites in metabolomics were mapped to the KEGG pathway for the biological interpretation of higher-level systemic functions. Finally, metabolites were considered statistically significant when their *p*-value was <0.05 and VIP value was >1.

All statistical analyses were conducted using SPSS software (version 26.0, IBM, Armonk, NY, USA). Categorical variables are described using percentages [n/(%)], and between-group comparisons were assessed using the *x*^2^ test. The measurement data require a prior normality test. For the measurement data that conform to the normal distribution, they are described in terms of mean ± standard deviation, and the comparison between the two groups was carried out using the *t*-test. In the case of data with a skewed distribution, they are denoted by the median (interquartile range), and the comparison between the two groups was implemented through the Mann-Whitney U test. Statistical significance was set at *p* < 0.05. Receiver operating characteristic (ROC) curves were employed to evaluate diagnostic accuracy.

## 3. Results

### 3.1 Characteristics of The Patients

**Fig. 1.**
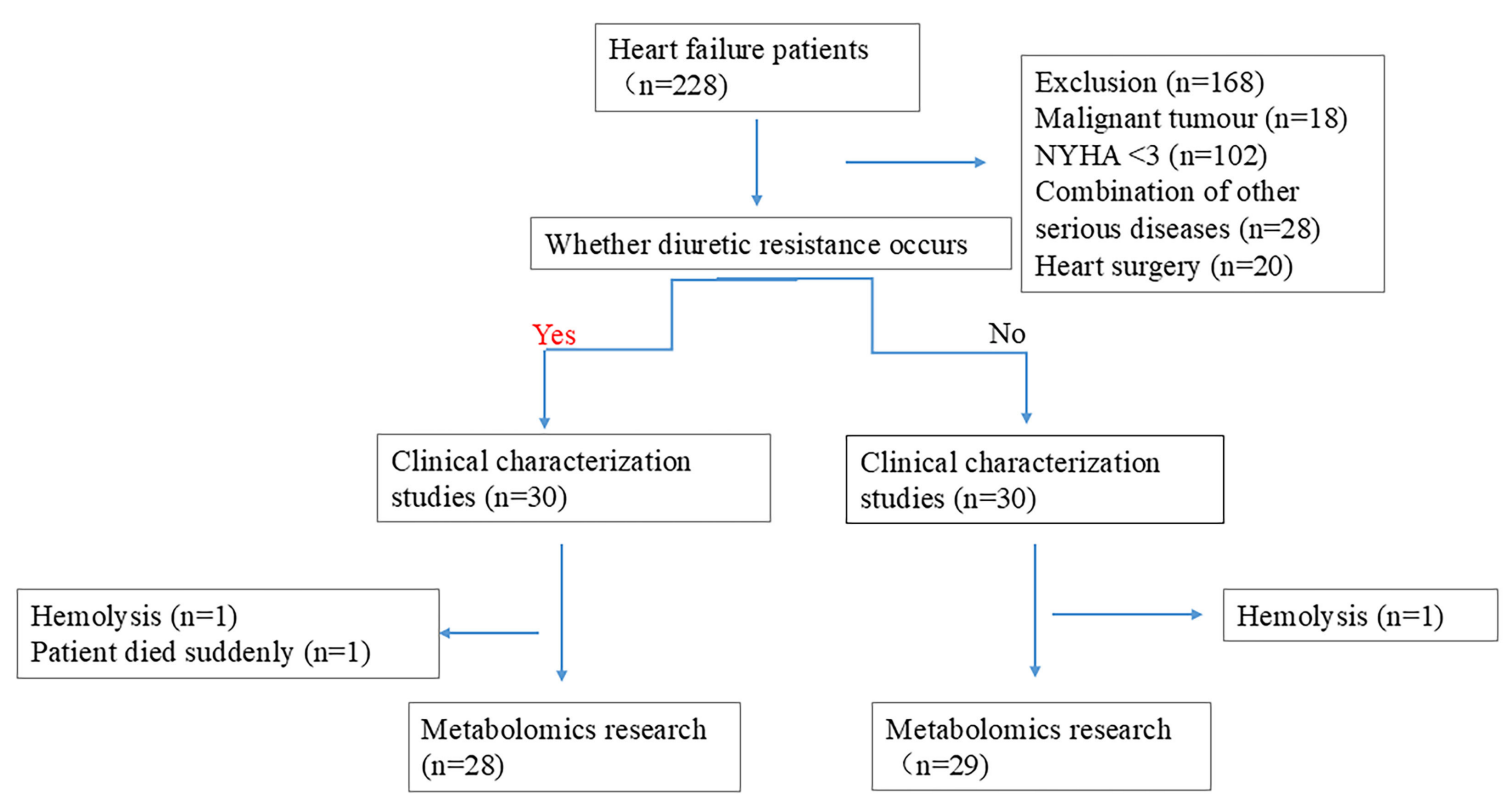
**Case inclusion flowchart**. NYHA, New York Heart Association.

This study included 60 participants, with 30 cases in the diuretic-resistant group and 30 cases in the non-diuretic-resistant group. From these participants, 57 serum samples were collected. Hemolysis occurred in two blood samples, and one patient died on the same day of blood collection due to severe electrolyte disturbances and multi-organ failure. This resulted in a total of 28 cases in the diuretic-resistant group and 29 cases in the non-diuretic-resistant group. The sampling procedure is illustrated in Fig. 1. The baseline characteristics of the 60 patients are summarised in Table 1. There were no significant differences in sex, age, heart rate, temperature, blood pressure, body mass index, NYHA functional class, smoking status, comorbidities or creatine between the diuretic-resistant and non-diuretic-resistant groups (*p* > 0.05). However, there were significant differences in N-terminal prohormone of brain natriuretic peptide (NT-proBNP) (*p* = 0.014) and eGFR (*p* = 0.047) between the two groups. The NT-proBNP level in the diuretic-resistant group was significantly higher than that in the non-diuretic-resistant group. Moreover, eGFR was negatively correlated with DR. The smaller the eGFR value, the greater the likelihood of DR occurrence.

**Table 1.** **Baseline characteristics of the patients**.

		Diuretic-resistant	Non-diuretic-resistant	*p *value
No. of case		30	30	
Age, years		72.0 ± 5.1	72.7 ± 4.0	0.577
Male, n/%		17/56.7	18/60.0	0.793
Heart rate, beats/min		79.9 ± 12.7	83.5 ± 21.7	0.435
Temperature, °C		36.47 (0.23)	36.46 (0.30)	0.861
Blood pressure, mm Hg				
	Systolic	123.8 ± 23.6	133.1 ± 25.5	0.150
	Diastolic	77.3 (17.0)	79.3 (18.0)	0.544
BMI (18.5–24.9), n/%		21/70.0	23/76.7	0.559
NYHA III, n/%		13/43.3	16/53.3	0.438
NT-proBNP, pg/mL		5666.1 (5446.1)	3233.2 (3594.7)	0.014
Smoke, n/%		11/36.7	9/30.0	0.584
Comorbidity				
	Coronary heart disease, n/%	21/70.0	16/53.3	0.184
	Cardiomyopathy, n/%	8/26.7	8/26.7	>0.999
	Atrial fibrillation, n/%	2/6.7	2/6.7	>0.999
History of cardiac surgery, n/%		6/20.0	1/3.3	0.108
Hypertension, n/%		21/70.0	20/66.7	0.781
Diabetes, n/%		14/46.7	12/40.0	0.602
Creatinine (umol/L)		106.84 ± 41.22	95.32 ± 32.10	0.232
eGFR (min × 1.73 m^2^)		62.35 ± 28.22	78.57 ± 33.40	0.047

BMI, body mass index; NT-proBNP, N-terminal prohormone of brain natriuretic peptide; eGFR, estimated glomerular filtration rate.

### 3.2 Metabolomic Analysis

#### 3.2.1 Quality Control 

**Fig. 2.**
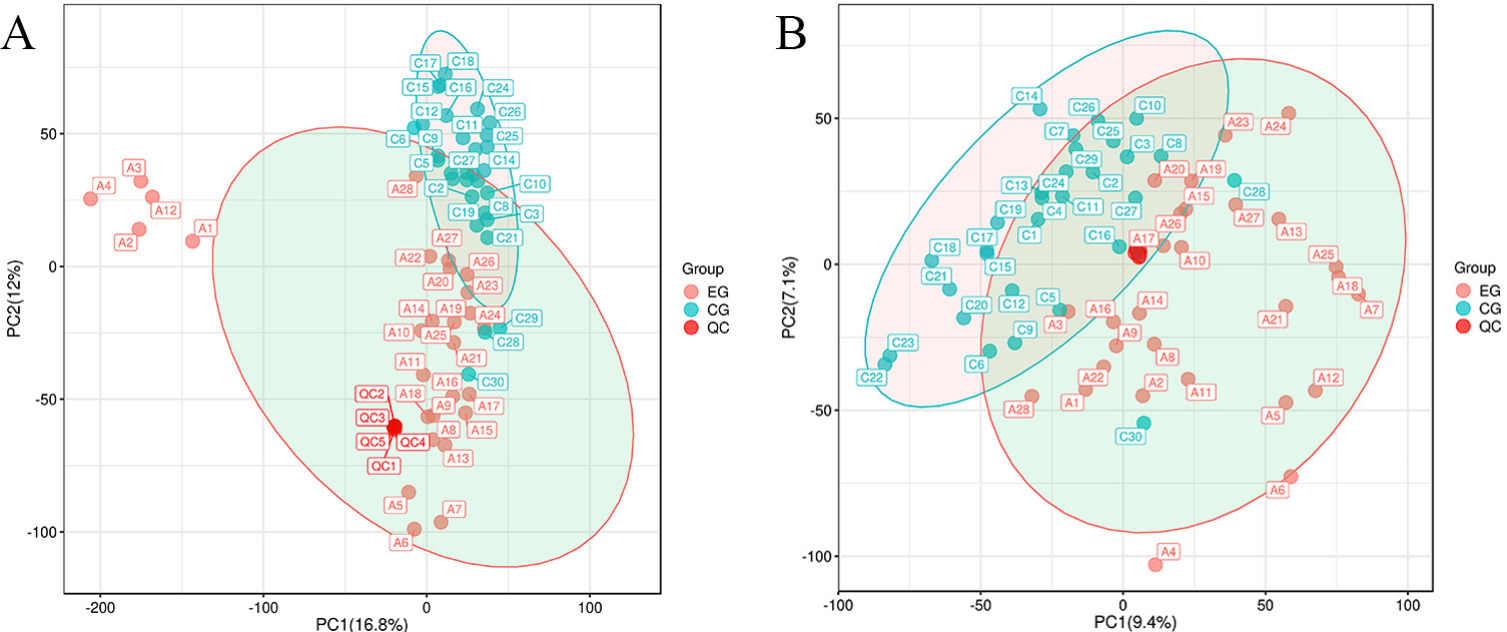
**PCA score chart for QC samples**. (A) Positive ion mode: graph of PCA scores for QC samples. (B) Negative ion mode: graph of PCA scores for QC samples. CG, non-diuretic-resistant group; EG, diuretic-resistant group; PCA, principal component analysis; QC, quality control; PC1, the first principal component; PC2, the second principal component.

In both ESI+ and ESI- modes, the PCA plot displayed tight clustering of QC samples across all samples (Fig. 2). This clustering suggests excellent analytical reproducibility and underscores the reliability of the findings in the current metabolomics study.

#### 3.2.2 Analysis of Differences between Groups 

**Fig. 3.**
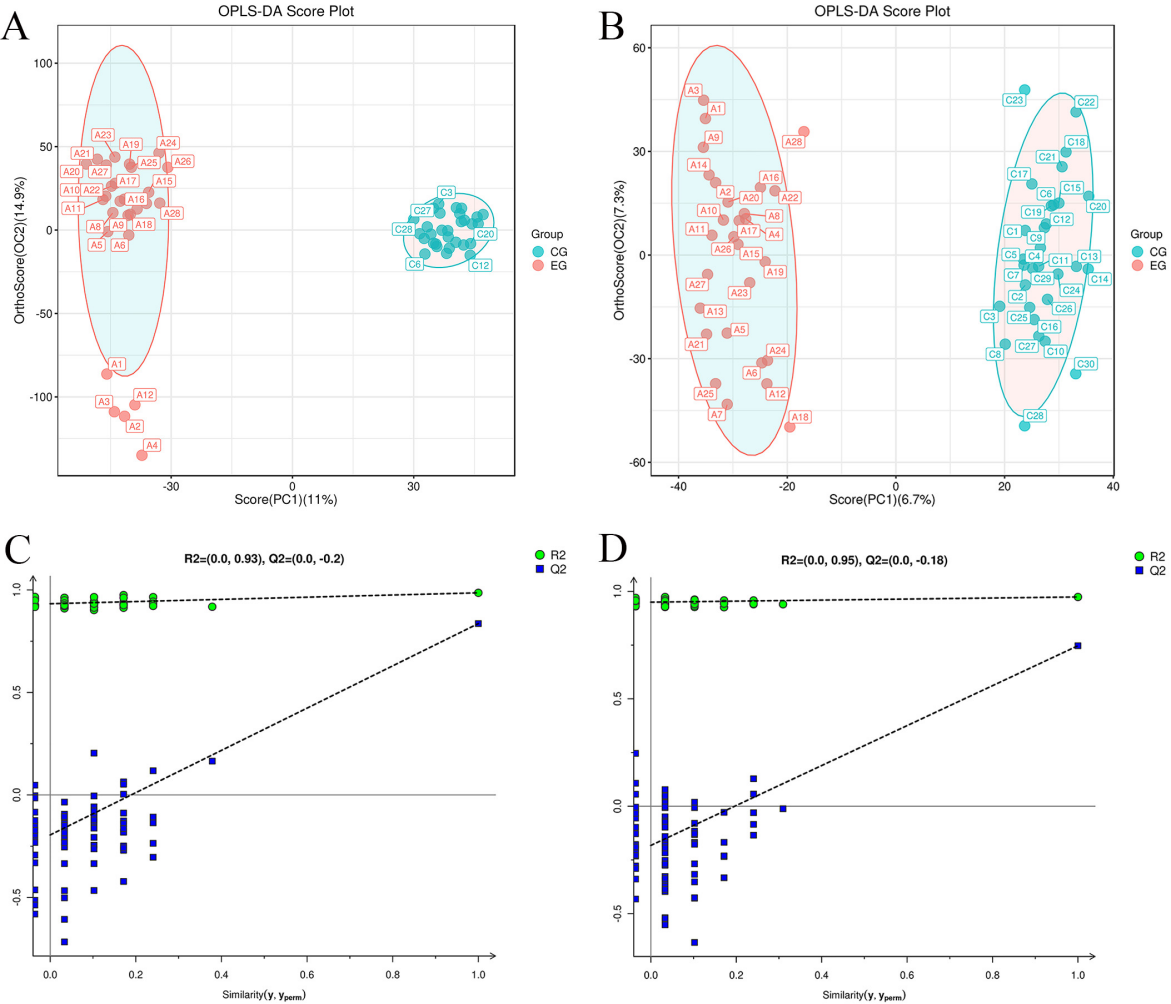
**Orthogonal partial least-squares discriminant analysis OPLS-DA scores and replacement test results for serum samples from both groups**. (A) Positive ion mode: OPLS-DA scores. (B) Negative ion mode, OPLS-DA scores. (C) Positive ion mode: OPLS-DA replacement inspection chart. (D) Negative ion mode: OPLS-DA replacement inspection. OPLS-DA, orthogonal partial least-square discriminant analysis; R2 and Q2 respectively refer to the values of the intersection points of the two regression lines R and Q with the y-axis.

Supervised metabolomics analysis was conducted using OPLS-DA. The score plot (Fig. 3A,B) depicted distinct clustering of samples within respective groups and dispersion of samples between groups, indicating reliable findings. Additionally, the permutation test plots (Fig. 3C,D) revealed that all blue Q2 points were situated below the original blue Q2 point on the far right, indicating the validity of the analysis.

#### 3.2.3 Correlation Analysis of Differentially Expressed Metabolites 

**Fig. 4.**
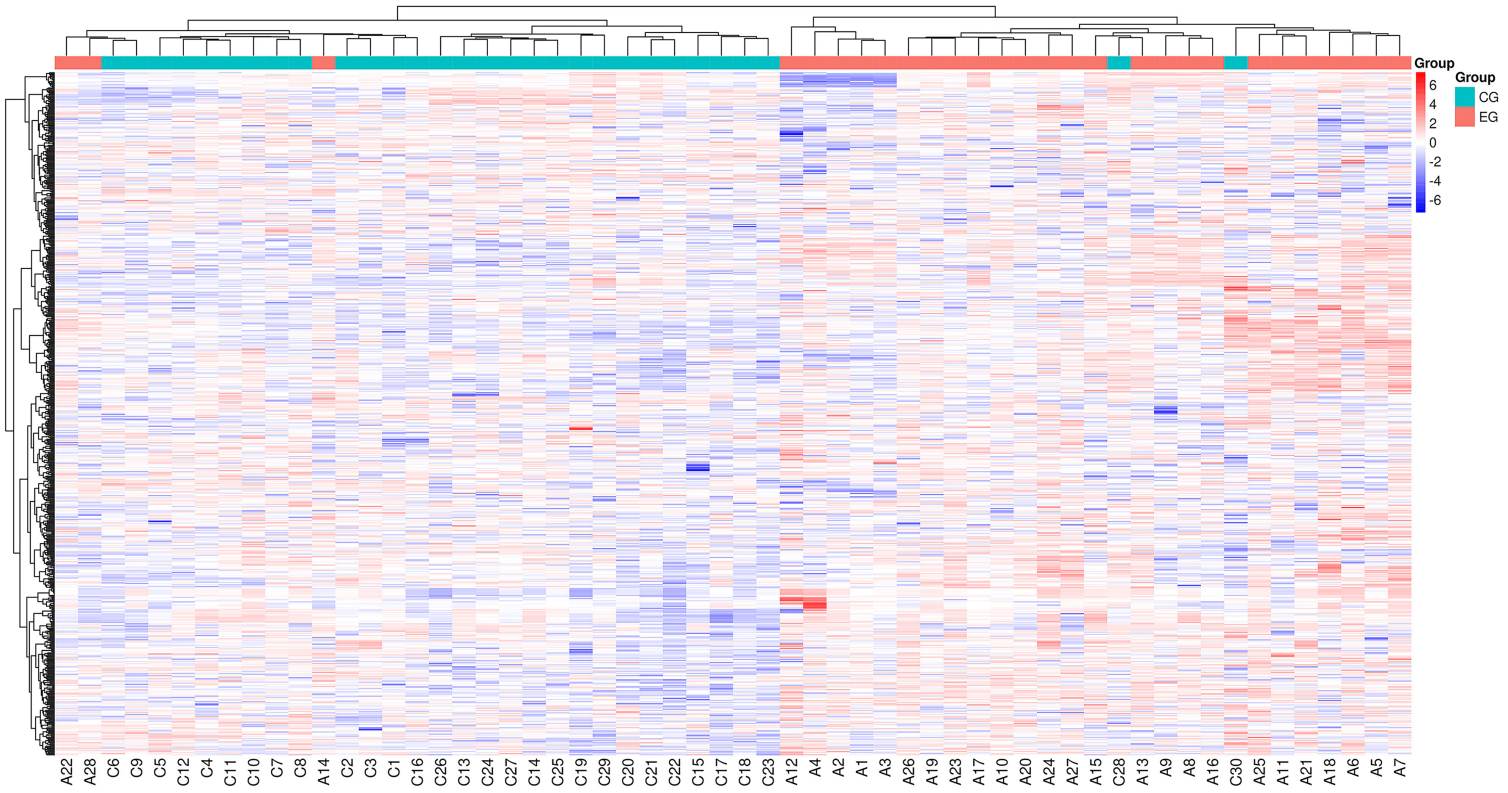
**Hierarchical clustering heat map of differential metabolites**. The color difference in the graph indicates the relative content. A redder color represents a higher expression, while a bluer color indicates a lower expression. The columns stand for the samples, and the rows represent the names of metabolites. The differential metabolite clustering tree is located on the left side of the graph. When the number of metabolites exceeds 150, their names will not be displayed.

**Fig. 5.**
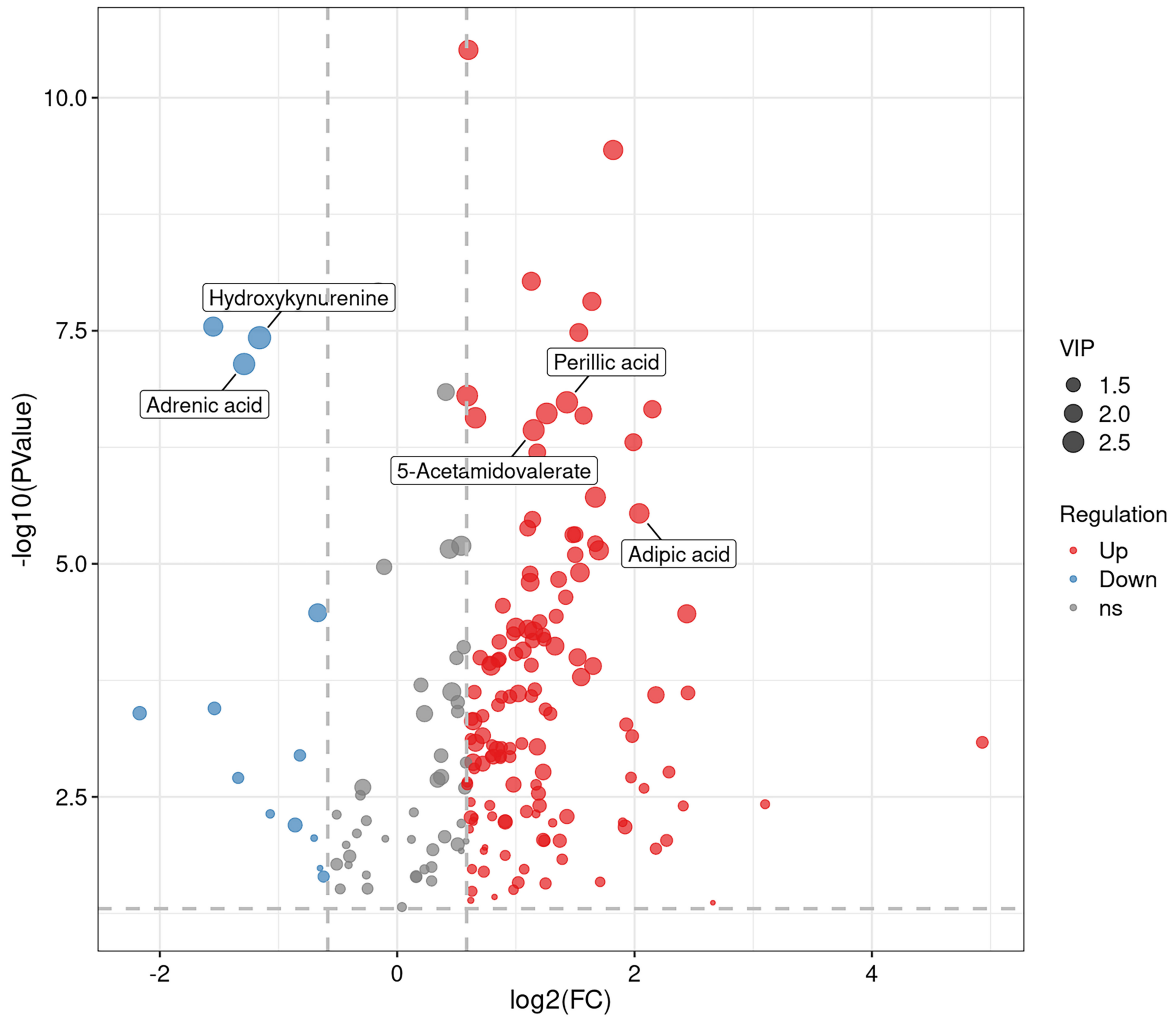
**Volcanic plot**. In the figure, each point stands for a metabolite. The x-axis represents the log2 value of the quantitative difference of a metabolite between two samples, while the y-axis represents the log10 value of the *p* value. A larger absolute value of the x-axis indicates a greater difference in the expression multiplicity of a metabolite between the two samples. A larger y-axis value indicates more significant differential expression, and the differentially expressed metabolites obtained through screening are more reliable. By default, the names of the top 5 metabolites with the smallest *p* values are displayed. ns refers to substances that have no significant difference.

Differential metabolites were identified from the list of sample-level substances and screened using a predefined threshold of *p*-value < 0.05 and VIP >1.0 in the statistical analysis. A total of 192 differential metabolites were detected, comprising 164 up-regulated and 28 down-regulated differential metabolites. Among them, the five most significant differential endogenous metabolites were Hydroxykynurenine, Perillic acid, Adrenic acid, 5-Acetamidovalerate, and Adipic acid (Table 2). These metabolites are presented in a hierarchical clustering heat map (Fig. 4) and volcanic plot (Fig. 5).

**Table 2.** **Major difference metabolites between diuretic-resistant and non-diuretic-resistant groups**.

Name	log2(FC)	*p* value	VIP	Tendency
Hydroxykynurenine	–1.16	3.75 × 10^-8^	2.758019	↓
Perillic acid	1.43	1.85 × 10^-7^	2.576167	↓
Adrenic acid	–1.29	7.19 × 10^-8^	2.565477	↓
5-acetamidovalerate	1.15	3.68 × 10^-7^	2.517805	↓
Adipic acid	2.04	2.88 × 10^-6^	2.242457	↓

VIP, variable importance on projection; FC, fold change.

#### 3.2.4 Defining Potential Biomarkers for the Early Diagnosis of DR

**Fig. 6.**
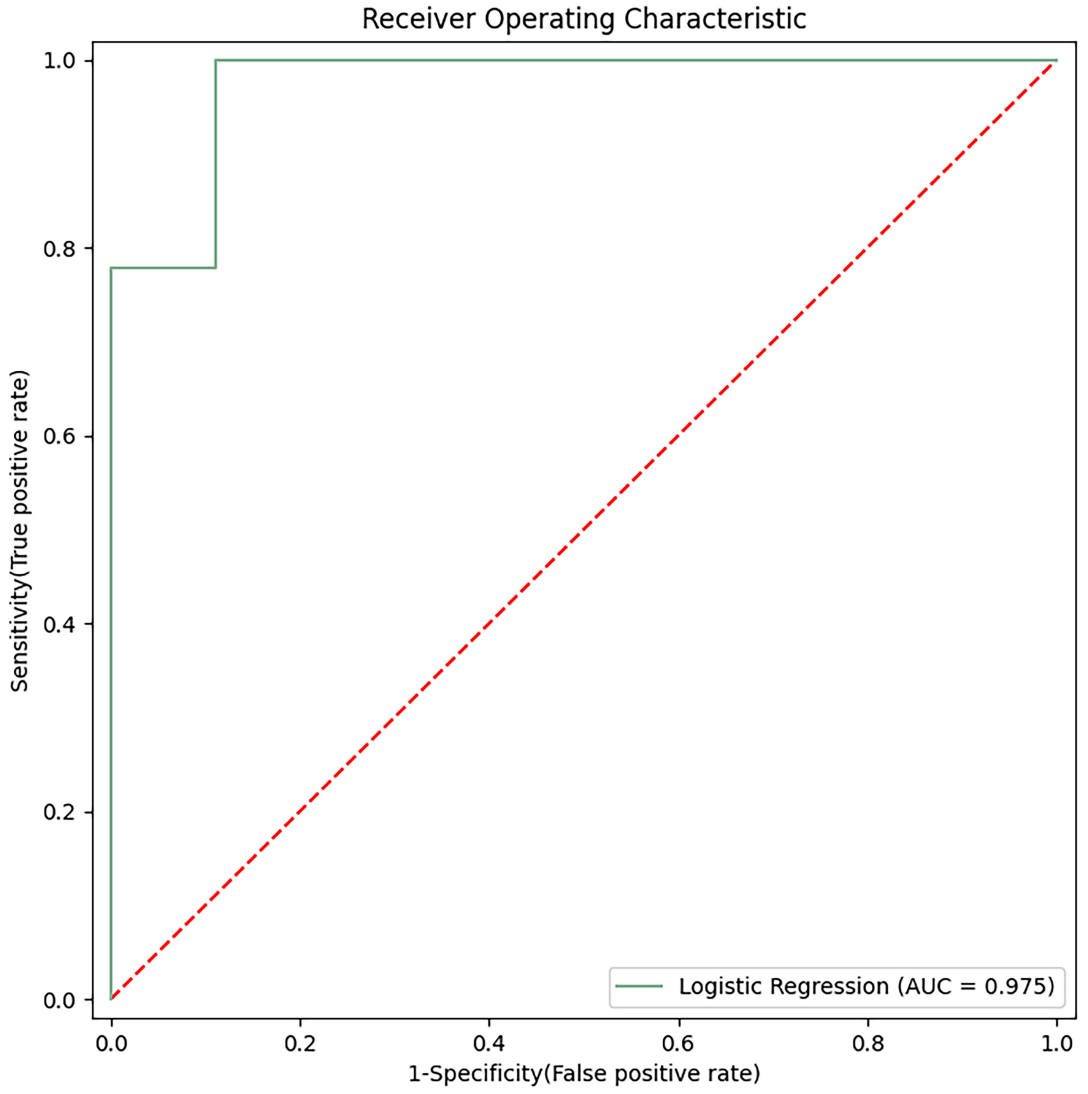
**Receiver operating characteristic analysis of the top five differential metabolites (hydroxykynurenine, perillic acid, adrenic acid, 5-acetamidovalerate, and adipic acid)**. AUC, area under curve.

To explore the predictive potential of these differential metabolites for DR, the top five metabolites showing significant differences, namely Hydroxykynurenine, Perillic acid, Adrenic acid, 5-Acetamidovalerate, and Adipic acid, were subjected to ROC analysis (Fig. 6). The area under the ROC curve was 0.975. These findings indicate that the top five significantly different metabolites hold the potential for early prediction of DR with favorable sensitivity and specificity.

#### 3.2.5 The Enriched Metabolic Pathways 

The KEGG database serves as a valuable resource for the systematic analysis of gene function and genomic information [34]. To determine the most important biochemical metabolic pathways and signal transduction pathways associated with the metabolites, the KEGG pathway enrichment analysis was performed on 192 different metabolites.

Under the criteria of *p* < 0.05 and pathway impact >0.1, indicative of significant pathway involvement, a total of 28 pathways were significantly affected (Table 3). The differential metabolites between the two groups were predominantly enriched in various pathways, including neuroactive ligand-receptor interaction, biosynthesis of amino acids, central carbon metabolism in cancer, alanine aspartate and glutamate metabolism, protein digestion and absorption, tyrosine metabolism, beta-alanine metabolism, phenylalanine metabolism, aminoacyl-transfer ribonucleic acid (tRNA) biosynthesis, glycine serine and threonine metabolism, arginine biosynthesis, valine/leucine and isoleucine biosynthesis, monobactam biosynthesis, mineral absorption, axon regeneration, cocaine addiction, lysine degradation, lysine biosynthesis, γ-aminobutyric acid (GABA) ergic synapse, amphetamine addiction, serotonin receptor agonists/antagonists, carbon fixation in photosynthetic organisms, cholesterol metabolism, alcoholism, insect hormone biosynthesis, cyclic adenosine monophosphate signalling pathway, phospholipase D signalling pathway, and prolactin signalling pathway.

**Table 3.** **Significant differences in metabolic pathways between the diuretic-resistant group and the non-diuretic-resistant group**.

Pathway_name	Total	*p* value	Impact
Neuroactive ligand-receptor interaction	52	3.333 × 10^-6^	0.912
Biosynthesis of amino acids	128	8.364 × 10^-6^	0.117
Central carbon metabolism in cancer	37	1.325 × 10^-5^	0.216
Alanine, aspartate and glutamate metabolism	28	1.691 × 10^-5^	0.250
Protein digestion and absorption	47	8.269 × 10^-5^	0.170
Tyrosine metabolism	78	1.314 × 10^-4^	0.128
beta-Alanine metabolism	32	3.815 × 10^-4^	0.189
Phenylalanine metabolism	60	4.765 × 10^-4^	0.133
Aminoacyl-tRNA biosynthesis	52	0.001	0.135
Glycine, serine and threonine metabolism	50	0.004	0.120
Arginine biosynthesis	23	0.005	0.174
Valine, leucine and isoleucine biosynthesis	23	0.005	0.174
Monobactam biosynthesis	39	0.007	0.128
Mineral absorption	29	0.012	0.138
Axon regeneration	7	0.018	0.286
Cocaine addiction	7	0.018	0.286
Lysine degradation	50	0.019	0.10
Lysine biosynthesis	35	0.022	0.114
GABAergic synapse	9	0.030	0.222
Amphetamine addiction	9	0.030	0.222
Serotonin receptor agonists/antagonists	1	0.031	1.00
Carbon fixation in photosynthetic organisms	23	0.033	0.130
Cholesterol metabolism	10	0.037	0.20
Alcoholism	10	0.037	0.20
Insect hormone biosynthesis	25	0.041	0.120
cAMP signaling pathway	25	0.041	0.120
Phospholipase D signaling pathway	11	0.044	0.182
Prolactin signaling pathway	11	0.044	0.182

tRNA, transfer ribonucleic acid; GABA, γ-aminobutyric acid; cAMP, cyclicadenosine monophosphate.

**Fig. 7.**
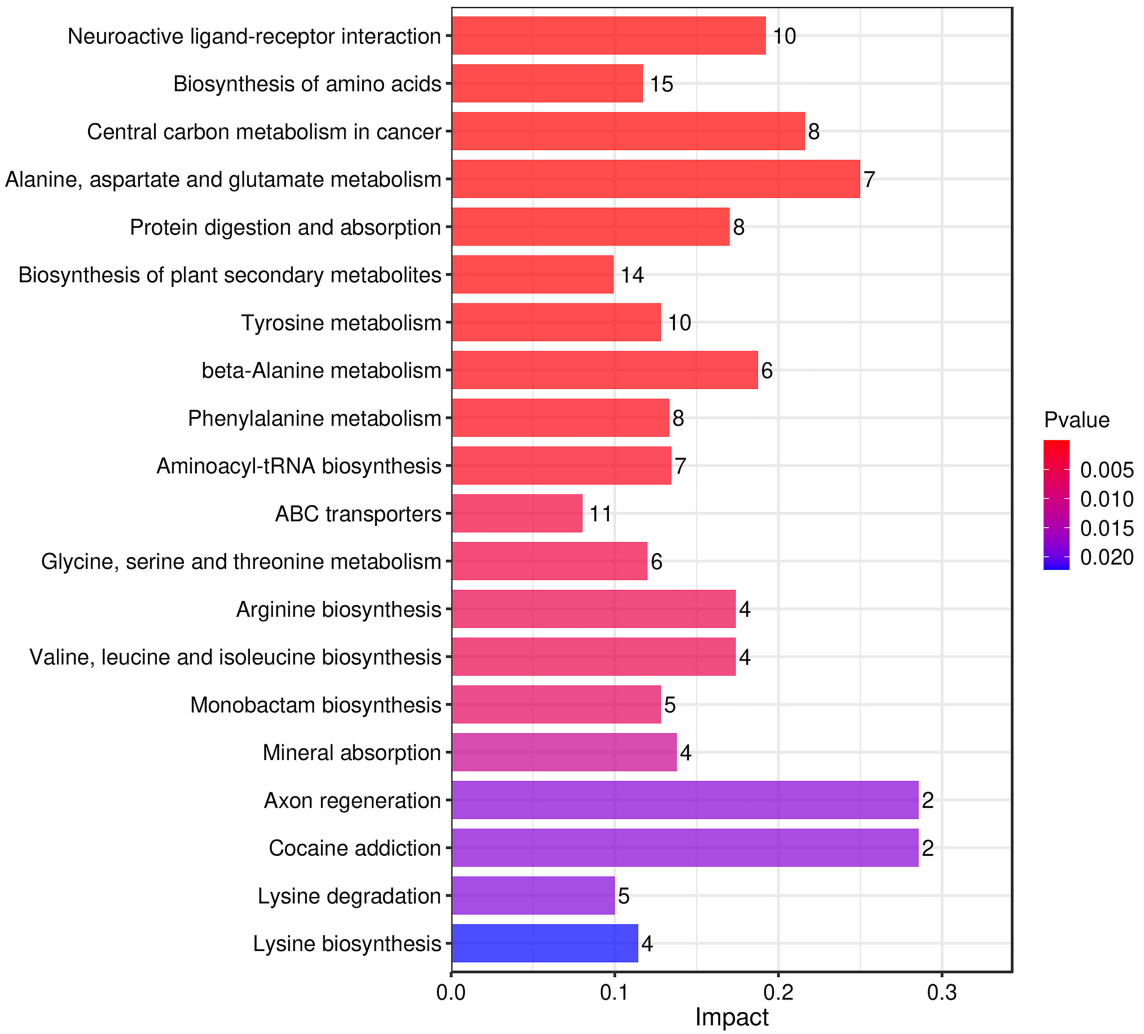
**Metabolic pathways influencing the factor histograms**. The vertical axis represents metabolic pathways, and the horizontal axis represents the Impact values enriched in different metabolic pathways. The higher the value, the greater the contribution of the metabolites detected under that pathway. The color is related to the *p*-value; the redder the color, the smaller the *p*-value, and the bluer the color, the larger the *p*-value. A smaller *p*-value indicates that the detected differential metabolites have a more significant impact on the pathway. ABC, adenosine triphosphate-binding cassette.

The KEGG enrichment histogram (Fig. 7) depicted the associated metabolic pathways. The most significantly different pathways between the diuretic-resistant and non-diuretic-resistant groups are neuroactive ligand-receptor interaction, biosynthesis of amino acids, and central carbon metabolism in cancer.

**Fig. 8.**
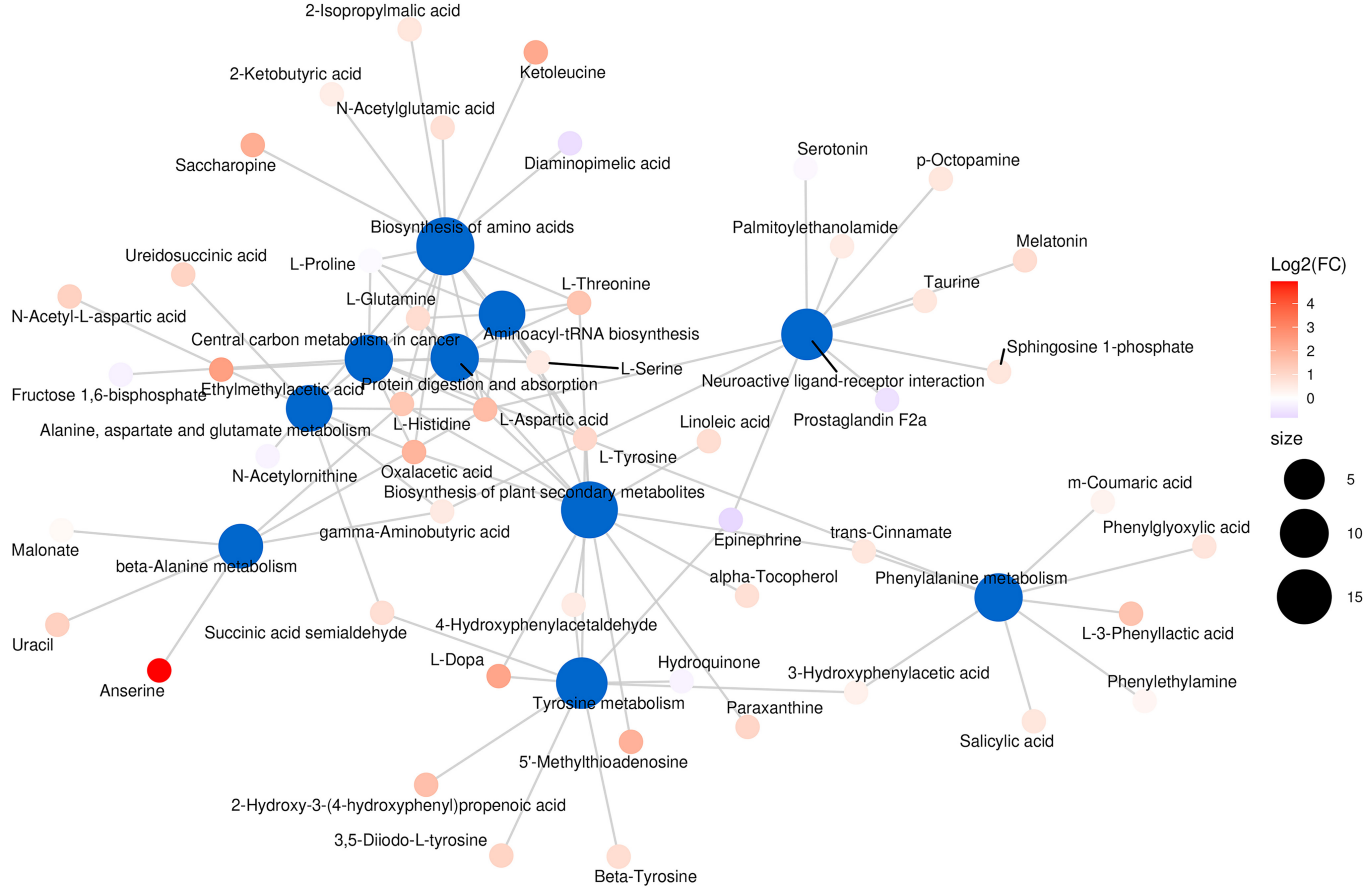
**Network diagram for the KEGG pathway enrichment analysis**. Circles in blue denote the pathways, whereas the other circles symbolize the metabolites. The magnitude of the pathway circles corresponds to the quantity of associated metabolites; the greater the number of metabolites, the larger the circle appears. The metabolite circles are shaded with a gradient to reflect the extent of the log2(FC) values, with no log2(FC) data presented for multiple comparisons. KEGG, kyoto encyclopaedia of genes and genomes.

Fig. 8 presents the metabolic pathway network diagram. The diagram highlights the pathways most enriched, including neuroactive ligand-receptor interactions, amino acid biosynthesis, and central carbon metabolism in cancer.

## 4. Discussion

This study employed untargeted metabolomics analysis via LC-MS/MS to identify 192 differential metabolites distinguishing between diuretic-resistant and non-diuretic-resistant groups. Among them, 164 metabolites were up-regulated and 28 were down-regulated. Notably, the top five significant differential endogenous metabolites were Hydroxykynurenine, Perillic acid, Adrenic acid, 5-Acetamidovalerate, and Adipic acid. The diagnostic potential of these five key metabolites was evaluated through ROC curve analysis, suggesting their promising utility as early markers for diagnosing the DR phynotype of HF. For example, in routine blood tests of patients, if the levels of these biomarkers can be monitored, when abnormal changes are detected, even if the patient has not yet exhibited traditional symptoms of DR, doctors can preliminarily determine that the patient is at a high risk of developing DR based on the changes in these biomarkers. During the treatment process, continuous monitoring of the levels of these biomarkers can reflect the effectiveness of the treatment plan in real time, enabling timely adjustment of the treatment plan.

In the KEGG analysis, the pathway demonstrating the most pronounced disparities was the neuroactive ligand-receptor interaction. This pathway exhibited a notable concentration of ligands and receptors on the plasma membrane, indicating a potential close association between the pathogenesis of DR and intracellular as well as extracellular ionic pathways and signal transduction.

Imbalances in various amino acids have been observed in diuretic-resistant and non-diuretic-resistant groups. Amino acids play vital roles in numerous cellular biosynthetic and metabolic processes, some of which have been linked to HF [38]. Additionally, amino acid metabolism is closely associated with the progression of DR. Among the dysregulated metabolites identified in metabolomics analyses, certain compounds exhibit neurotransmitter properties or neuroactive functions. For instance, GABA, a prominent inhibitory neurotransmitter in the nervous system, acts by binding to specific transmembrane receptors on the plasma membrane of both pre- and postsynaptic neurons. Alterations in GABAergic input to the paraventricular nucleus in patients with chronic HF maintain a sympathetic vasodilatory tone. Elevated GABA levels might offer novel insights into the neurological influences on disease progression in HF [39]. However, further biological experiments are warranted to elucidate the detailed mechanisms involved. Furthermore, several metabolites categorised as dipeptides have been identified as dysregulated from the stage of HF to DR. Examples include prolylhydroxyproline, glutamylphenylalanine, and threonylleucine. These compounds are typically regarded as breakdown products of protein digestion or proteolytic metabolism, with some dipeptides serving physiological or cellular signalling functions [40]. Given the presence of cardiac structural alterations spanning from HF to DR, these dipeptides might stem from protein digestion within abnormal cardiac cells, holding promise as potential diagnostic biomarkers.

Differences were also observed in central carbon metabolism in cancer between the two groups, suggesting a potential link between severe HF and cancer. First, factors such as heightened oxidative stress, low-level inflammatory response, activation of the neurohormonal system, and immune system dysregulation might collectively contribute to the development of HF and cancer. Second, alterations in the cardiac extracellular matrix influence tumour stroma. As HF progresses, the stroma undergoes significant changes, becoming more fibrotic [41, 42]. This shift in the microenvironment not only precipitates pathological changes such as cardiomyocyte hypertrophy and abnormal energy metabolism but also indirectly stimulates other organs, including tumor tissues, via the bloodstream. This stimulation occurs through the release of paracrine or endocrine growth factors, cytokines, and chemokines. Therefore, future studies can explore HF biomarkers based on those associated with cancer.

There are differences in the pathways associated with protein digestion and absorption between the two groups. Patients with HF often experience concomitant digestive and absorption dysfunction, resulting in inadequate protein intake and various adverse effects such as hypoproteinaemia [43, 44]. Consequently, hypoproteinaemia limits the ability of diuretics by impeding their ability to reach appropriate concentrations at their target sites, including distal tubular adaptation, among others. Aminoacyl-tRNA plays a pivotal role in shuttling amino acids to ribosomes for protein synthesis [45, 46]. Aminoacyl-tRNA synthetases (ARSs) are widely distributed in organisms. Disturbances in aminoacyl-tRNA biosynthesis observed in patients with DR might be associated with disturbances in the metabolism of numerous amino acids. Several mutations might result in compromised aminoacylation or editing activity or altered gene expression levels of ARSs [47]. The adenosine triphosphate-binding cassette (ABC) transporter protein family comprises crucial efflux-type transporter proteins in the human body, with P-glycoprotein, breast cancer resistance protein, and multidrug resistance protein representing prominent members [48]. The administration of diuretics in various clinical diseases can impact the function or expression of ABC transporter proteins, subsequently affecting the *in vivo* dynamic processes and efficacy of co-administered chemotherapeutic drugs.

Apart from these identified differential metabolites and metabolic pathways, traditional markers are of great significance in DR. The pathophysiological mechanism underlying DR involves complex interactions at multiple levels. From the perspective of the cardiorenal axis, a reduction in cardiac output leads to insufficient renal perfusion and a gradual impairment of renal function. Some clinical guidelines recommend continuous measurement of natriuresis in patients with acute heart failure (AHF) to monitor DR [49]. A previous study has shown that long-term use of loop diuretics leads to a weakened natriuretic response [50]. The reasons are as follows: first, the relative or absolute reduction in extracellular fluid volume reduces the transport of solutes to the proximal renal tubules through the mechanisms mediated by the RAAS and the sympathetic nervous system; second, long-term exposure to loop diuretics induces adaptive epithelial hypertrophy and hyperfunction in the distal renal units, manifested as hypertrophy of distal tubular cells [51]. This structural change in cells leads to a compensatory increase in sodium reabsorption, alters the renal response to diuretics, and ultimately results in a decrease in blood sodium levels during chronic loop diuretic treatment [52]. Clinicians should closely monitor the renal function of long-term users and adjust the use of diuretics accordingly.

In the in-depth study of metabolic changes in HF patients, the liver dysfunction in AHF and its metabolic impact are significant. Research [53] indicates that AHF patients’ hepatorenal dysfunction is complex. At admission, 82% had an elevated Model of End-Stage Liver Dysfunction (MELD)-XI score, with the prevalence of different levels of dysfunction changing over time. Hepatorenal-dysfunction patients have unique clinical features, and the MELD-XI score-prognosis correlation shows the importance of liver function in disease progression. Pathophysiologically, liver congestion causes liver dysfunction, affecting drug metabolism, disrupting metabolic balance via abnoral kidney interactions, and potentially impacting amino acid, nucleotide, and energy metabolism. MELD-XI in AHF is crucial for risk stratification and treatment decisions related to liver and kidney function, and is predictive of treatment response. In view of this, the differential metabolites in our study may be affected by liver dysfunction, so their specificity and accuracy as DR biomarkers need further verification. Future research should more comprehensively assess liver function.

In patients with HF, accurately assessing mortality risk and assessing disease severity are crucial for effective clinical management and targeted interventions. DR frequently precipitates hospitalisation due to congestion and exacerbation of symptoms, although it is sometimes preventable or reversible. A recent study suggests that urine sodium can be used to identify patients with DR, and it may play an important role in guiding individual treatment [54, 55]. However, the real clinical value for patients with DR remains to be investigated. Hence, the development of novel diuretics, strategies, or combinations is imperative to overcome DR.

### Study Limitations

Our study has some limitations. First, the sample size of patients with HF in this study was small, and all participants were from Changsha, China, potentially introducing geographical biases in the results. Second, our study is exploratory and preliminary, a larger cohort to exclude patients undergoing other treatments is necessary before its applicability in clinical practice can be ascertained. Additionally, this study did not provide detailed information on the medications taken by patients, making it impossible to estimate the impact of these medications on the metabolite levels in HF patients. Furthermore, we did not exclude metabolic diseases related to HF, which might have a certain influence on the metabolomics results. Finally, it cannot be conclusively determined whether the identified differential metabolites in this study are specific to DR patients. In the future, animal and clinical validation are still required to render the entire research more comprehensive.

## 5. Conclusions

We used an untargeted LC-MS/MS metabolomics approach to analyze blood metabolites from HF patients and identify metabolic differences between diuretic-resistant and non-diuretic-resistant cases. Key differential metabolites included Hydroxykynurenine, Perillic acid, Adrenic acid, 5-Acetamidovalerate, and Adipic acid. Significant metabolic pathways that were affected included amino acid, energy, and nucleotide metabolism. Further evidence-based diagnostic testing is needed to fully understand the roles of these pathways in DR. This study highlights the potential of non-targeted metabolomics in improving the identification and management of DR in patients with HF.

## Availability of Data and Materials

All data involved in the article have been submitted as part of the results section. All data reported in this paper will also be shared by the lead contact upon request.

## Supplementary Material

Supplementary material associated with this article can be found, in the online version, at https://doi.org/10.31083/RCM27001.
