# Supplementary material for: Application of Metabolomics and the Discovery of Potential Serum Biomarkers for Diuretic Resistance in Heart Failure
Source: Rev Cardiovasc Med. 2025 Apr 22;26(4):27001. doi: 10.31083/RCM27001 (PMC12059756; doi:10.31083/RCM27001)
Supplement: Supplementary file 1 [file 2153-8174-26-4-27001-s1.docx]

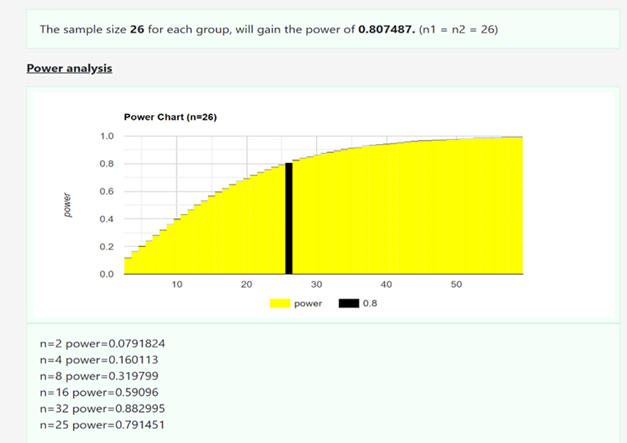


**Supplemental File 1** Sample size calculation results


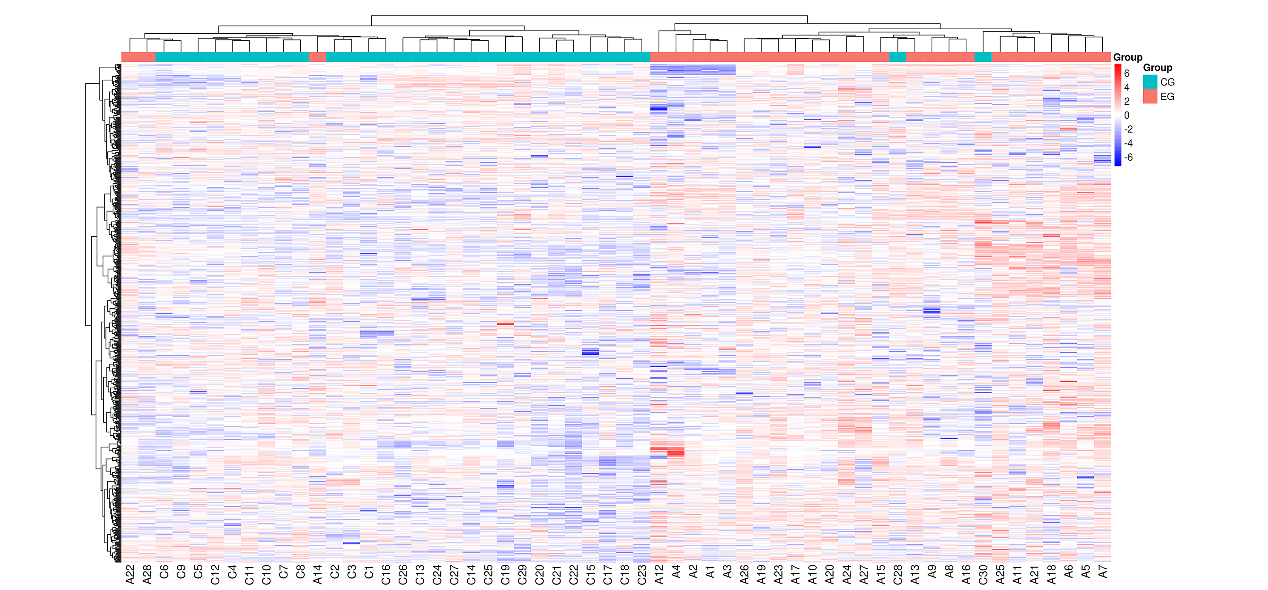


Supplemental File 2 Hierarchical clustering heat map of differential metabolites

The relative content of the graph is shown by the color difference, the redder the higher the expression, the bluer the lower the expression. The columns represent the samples, the rows represent the metabolite names, the clustering tree on the left side of the graph is the differential metabolite clustering tree, and the metabolite names are not shown when the number of metabolites exceeds 150.


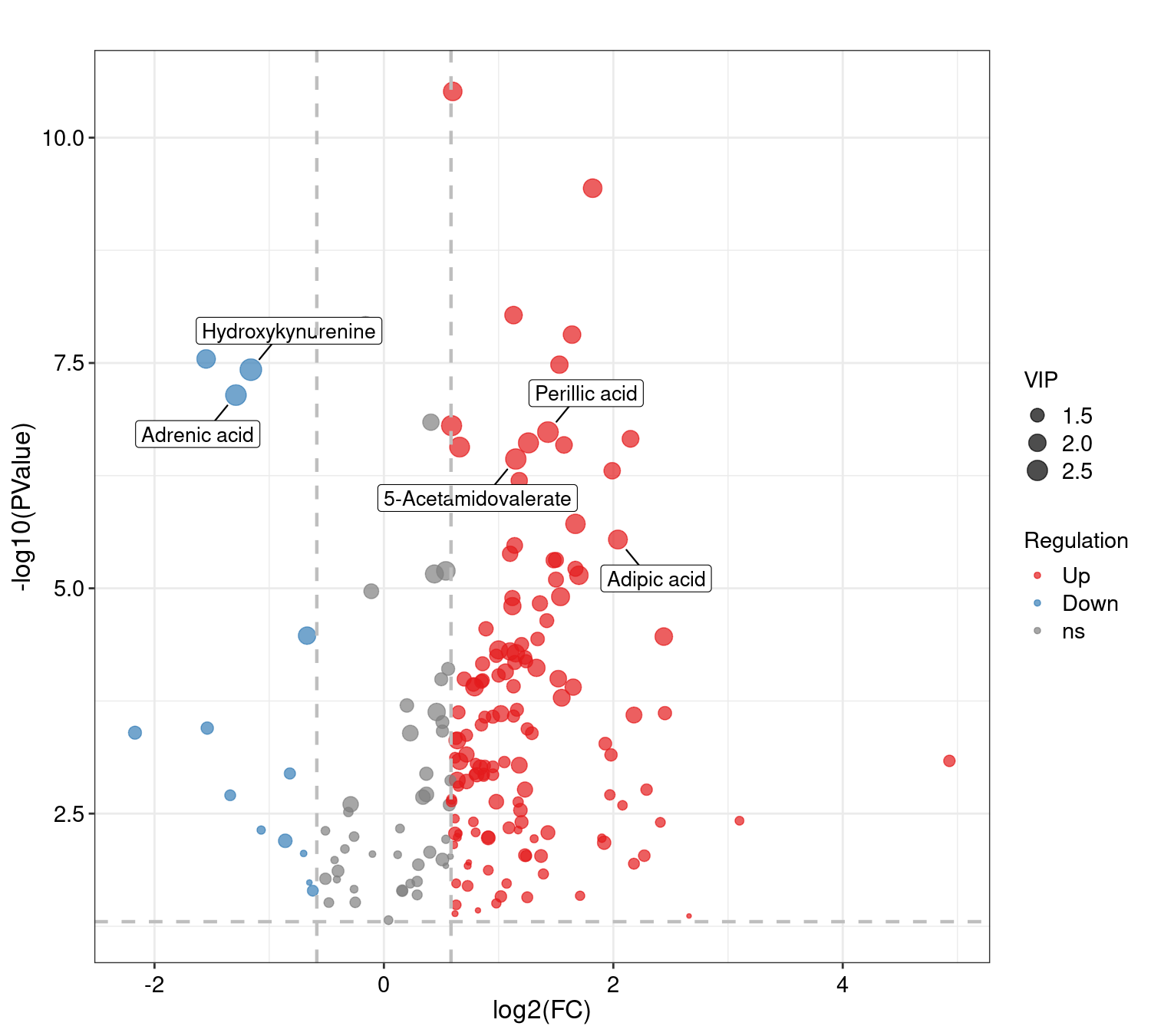


Supplemental File 3 Volcanic plot

Each point in the figure represents a metabolite, and the horizontal coordinate represents the log2 value of the quantitative difference of a metabolite between two samples; the vertical coordinate represents the log10 value of the P value. A larger absolute value of the horizontal coordinate indicates a larger difference in the multiplicity of expression of a metabolite between the two samples. The larger value of the vertical coordinate indicates the more significant differential expression and the more reliable differentially expressed metabolite obtained by screening. The red points represent up-regulated differentially expressed metabolites, the blue points represent down-regulated differentially expressed metabolites, and the gray points represent metabolites that were detected but did not meet the filtering parameters. The top 5 metabolite names with the smallest P value are displayed by default.
